# Neonatal Coronavirus 2019 (COVID-19) Infection: A Case Report and Review of Literature

**DOI:** 10.7759/cureus.8165

**Published:** 2020-05-17

**Authors:** Vikramaditya Dumpa, Ranjith Kamity, Alexandra N Vinci, Estela Noyola, Asif Noor

**Affiliations:** 1 Pediatrics, New York University (NYU) Winthrop Hospital, Mineola, USA; 2 Pediatrics, New York University (NYU) Long Island School of Medicine, Mineola, USA

**Keywords:** covid-19 in neonates, sars-cov-2, transmission in neonatal covid-19

## Abstract

Coronavirus disease 2019 (COVID-19), caused by severe acute respiratory syndrome coronavirus 2 (SARS-CoV-2), has led to a global pandemic affecting 213 countries as of April 26, 2020. Although this disease is affecting all age groups, infants and children seem to be at a lower risk of severe infection, for reasons unknown at this time. We report a case of neonatal infection in New York, United States, and provide a review of the published cases. A 22-day-old, previously healthy, full-term neonate was hospitalized after presenting with a one-day history of fever and poor feeding. Routine neonatal sepsis evaluation was negative. SARS-CoV-2 polymerase chain reaction (PCR) testing was obtained, given rampant community transmission, which returned positive. There were no other laboratory or radiographic abnormalities. The infant recovered completely and was discharged home in two days once his feeding improved. The family was advised to self-quarantine to prevent the transmission of COVID-19. We believe that the mode of transmission was horizontal spread from his caregivers. This case highlights the milder presentation of COVID-19 in otherwise healthy, full-term neonates. COVID-19 must be considered in the evaluation of a febrile infant. Infants and children may play an important role in the transmission of COVID-19 in the community. Hence, with an understanding of the transmission patterns, parents and caregivers would be better equipped to limit the spread of the virus and protect the more vulnerable population.

## Introduction

The novel severe acute respiratory syndrome coronavirus 2 (SARS-CoV-2), responsible for the coronavirus disease 2019 (COVID-19), emerged in Wuhan, China, in December 2019. It surged in the Lombardi region of Italy in February 2020 and, later, New York, USA, became the epicenter in March-April 2020. Severe COVID-19 is disproportionately affecting the elderly and people with underlying medical problems. In a review by the Chinese Center for Disease Control and Prevention, out of the 1,321 children tested positive for COVID-19, 31 (18.1 %) were less than one year of age [1]. Disease characterization of early cases among children in the United States revealed 398 (15%) cases under one year of age [2]. Separate data on cases in neonates is not available in these studies. Reports of neonatal cases are sparse, posing many unanswered questions on the disease characteristics of COVID-19 in this population.

## Case presentation

We report a three-week-old neonate with a COVID-19 infection in New York, USA. This 22-day-old male neonate presented to the emergency department on March 20, 2020, with a one-day history of fever and decreased oral intake. There was no history of cough or rhinorrhea. The parents were asymptomatic; however, the grandparents who visited the baby a week prior to his illness subsequently developed fever and cough two days after their visit. The infant was born via vaginal delivery at 39 weeks gestation, without complications. The mother had an uneventful antenatal course. The baby was discharged home with his mother two days after birth. He was exclusively breastfed and was healthy until this illness.

Physical examination on admission revealed a well-appearing baby in no acute distress. His rectal temperature was 100.7° F (38.1 °C). He was also tachycardic but the rest of the vital signs were normal. Initial laboratory evaluation included a white blood cell count of 4,000 cells /mm^3^ (normal range 7800-15,900 cells/mm^3^) with 17% neutrophils, 51% lymphocytes, 24% monocytes, and 1% immature neutrophils. Table 1 shows the patient characteristics, vital signs on admission, and laboratory values during the hospital course. In light of the community transmission of COVID-19, a nasopharyngeal swab was sent for SARS-CoV-2 PCR testing. The infant was kept in a negative pressure room with enhanced infection control precautions requiring an N95 mask, eye shield, gloves, and gowns. He was started on antibiotics after obtaining blood, urine, and cerebrospinal fluid for bacterial cultures.

**Table 1:** Patient characteristics, vital signs on admission, and laboratory values PCR: Polymerase chain reaction, SARS-CoV-2: Severe acute respiratory syndrome coronavirus 2

Patient Characteristics	
Gestational age	39 weeks
Age at presentation	22 days
Gender	Male
Mode of delivery	Vaginal
Resuscitation at delivery	None
APGARS (1 & 5 min)	9,9
Vital signs on admission	
Temperature (rectal)	100.7 °F
Heart rate	182/min
Respiratory rate	36/min
Blood pressure	86/48 mmHg
Oxygen saturation	98%
Laboratory values	
Complete blood count	
Hemoglobin (g/dL)	14.3
White blood cells (x 10^3^/µL)	4
Neutrophils (%)	17
Lymphocytes (%)	51
Platelets (x 10^3^/µL)	270
Cerebrospinal fluid analysis	
Red blood cells (/µL)	1670
White blood cells (/µL)	3
Glucose/protein (mg/dl)	45/76
Meningitis panel (PCR)	Negative
Culture	Negative
Urine analysis	Negative
Urine culture	Negative
Blood culture	Negative
Respiratory viral panel (PCR)	Negative
SARS-CoV-2 (PCR)	Detected

The infant remained hemodynamically stable and did not require any supplemental oxygen support. He was afebrile for the remainder of the hospitalization, with stable vital signs. The SARS-CoV-2 testing done by polymerase chain reaction assay at the New York State Wadsworth Center returned positive the day after the admission. The antibiotics were discontinued two days later when the bacterial cultures did not show any growth. The patient was discharged after two days of hospitalization with instructions on infection control at home when taking care of this infant, including meticulous hand hygiene, particularly before and after feeding and diaper changes. The family was also advised to self-quarantine to prevent the transmission of COVID-19. The infant continues to be healthy and thriving well at the four-week follow-up.

## Discussion

After the first case of COVID-19 was detected in New York on March 1, 2020, there continues to be a rapid increase in the number of infections and deaths in the ensuing weeks. As of April 26, 2020, there were 288,045 positive cases in the state of New York. There were 16,966 COVID-19-related deaths reported with two fatalities in children younger than 10 years (further details on mortality not available). We describe a case of neonatal COVID-19 infection during the early weeks of recognized widespread community transmission in New York. To the best of our knowledge, this is one of the first few reported neonatal cases of COVID-19 infection in the United States.

SARS-CoV-2, belonging to the genus Betacoronavirus, is a single-stranded ribonucleic acid (RNA) virus. The virus is transmitted across humans, primarily through respiratory droplets and contact. Infants and children affected by this virus usually have a history of exposure to a sick contact. There is no clear evidence of vertical transmission despite some reports suggesting this possibility in neonates [3-5]. The median incubation period is five days with a range of two to 14 days [6].

To date, there are 11 published cases of neonatal COVID-19 detected by SARS-CoV-2 PCR [3-5,7-11]. Six of them presented within three days of life whereas the others presented between five and 28 days of life. Seven out of the 11 neonates had fever as the presenting complaint. Eight of the neonates were born to mothers who tested positive for COVID-19. The clinical characteristics of eight infants, along with the infant described in the current report, are summarized in Table 2. Complete details on the other three cases were not available and hence not included in the table. Three separate neonates with COVID-19 presenting within the first two days of life were also reported in media, complete details of which were not available. Serological evidence of COVID-19 with elevated immunoglobulin-M levels was shown in three neonates but with negative PCR testing [12-13]. There is a lack of definitive evidence pointing towards in utero SARS-CoV-2 transmission at this time [14].

**Table 2:** Clinical characteristics of neonatal cases of coronavirus disease 2019 described thus far in English literature CS: Cesarean section, NP: Nasopharyngeal swab, PCR: Polymerase chain reaction, SARS-CoV-2: Severe acute respiratory syndrome-coronavirus 2

Author (Reference)	Zeng et al. (3)	Zeng et al. (3)	Zeng et al. (3)	Kamali et al. (7)	Patek et al. (10)	Coronado et al. (11)	Alzamora et al. (4)	Hu et al. (5)	Current case
Gestational age (Weeks)	40	40 4/7	31 2/7	Full-term	39	36	33	40	39
Day of life when illness started	2	0	0	15	14	21	0	Asymptomatic	22
Mode of delivery	CS	CS	CS	CS	CS	Not available	CS	CS	Vaginal
Presentation	Fever, lethargy	Fever, lethargy, vomiting	Respiratory distress	Fever, tachypnea, and mottling	Fever, erythema of digits, poor feeding	Congestion, tachypnea, reduced feeding	Respiratory insufficiency (?secondary to maternal general anesthesia)	N/A	Fever, decreased oral intake
Abnormal labs	Abnormal Procalcitonin	Leukocytosis, lymphopenia, elevated creatine kinase	Abnormal coagulation profile	None	Neutropenia, elevated liver enzymes, MRSA from wound cultures	Rhinovirus positive, elevated CRP, Procalcitonin	N/A	None	None
Chest radiography	Pneumonia	Pneumonia	Pneumonia	Normal	Bilateral perihilar streaking	Bilateral infiltrates	Normal	Normal	Normal
SARS-CoV-2 testing	NP, rectal swabs + PCR	NP, rectal swabs + PCR	NP, rectal swabs + PCR	NP + PCR	NP + PCR	NP + PCR	NP + PCR	Throat swab + PCR	NP + PCR
Probable mode of transmission	? Vertical	? Vertical	? Vertical	Horizontal	Horizontal	Horizontal	?Vertical	? Vertical	Horizontal
Outcome	Recovered	Recovered	Recovered	Recovered	Recovered	Recovered	Recovered	Recovered	Recovered

There was no reported breakdown data on neonates in the Centers for Disease Control and Prevention (CDC) Morbidity and Mortality Weekly Report from April 6, 2020. It indicated that only 1.7 % (2,572 of 149,082) of all COVID-19 infections in the USA occurred in patients under 18 years [2]. Infants accounted for 15% of the cases (393 of 2,572) among children under 18 years, comparable to 11.8% in the study by Dong et al. [15]. The common clinical manifestations of fever, cough, or shortness of breath were observed only in 73% of the cases, indicating that children may not always present with the classic symptoms, unlike adults. The severity of illness and the rates of hospitalization were disproportionately higher among infants in the above studies. Fever appears to be the main presenting symptom of COVID-19 in a majority of the neonates described in the literature so far (8 of 12, 66%) and the prognosis seems to be good [3-5,7-11]. In summary, even though infants appear to be at a higher risk of severe illness, neonates tend to have a milder infection based on the very limited number of cases published so far.

Data from large studies involving adult cases of COVID-19 indicate that the frequent laboratory abnormalities seen in these patients include lymphocytopenia, thrombocytopenia, leukopenia, and elevated inflammatory markers [16]. Laboratory abnormalities of neonatal COVID-19 patients discussed in this review are available in Table 1. It remains to be seen from larger studies if infected neonates mount the same response as adults, and if the response pattern is the same in neonates presenting early versus late.

Thus, based on the limited data available so far, we postulate that neonatal infection can be early-onset (first three days after birth) or late-onset (4-28 days of life). The majority of the cases are due to horizontal spread. At this time, data suggesting the possibility of vertical transmission is equivocal [5,14,17]. Data on serological testing from larger cohorts will give more insight into the transmission patterns. There is no evidence that this infection can be transmitted through breast milk [17].

There are several hypotheses explaining the reason for neonates being at lower risk for severe COVID-19. One hypothesis is related to the maturation and functioning of receptors. Angiotensin-converting-enzyme 2 has been proven to be the principal target of the SARS-CoV-2 virus. In neonates, it is possible that the receptor activity is immature or there is an increase in angiotensin-converting-enzyme 2 activity [18]. Another postulation is a higher CD4 to CD8 ratio in neonates. It has been observed that older males with COVID-19 infection had a low CD4 count. Infants, on the other hand, have a high CD4 to CD8 ratio [19]. This change in lymphocyte proportion is related to the changes and involution of thymus in infants. The protective role of maternal antibodies acquired transplacentally is also to be studied. There are also reports of thrombotic complications contributing to the mortality and morbidity in adult COVID-19 patients [20]. If this is true, the possible role of fetal hemoglobin in the milder presentation of COVID-19 in neonates needs to be further explored.

The management of a severe COVID-19 infection is still evolving. Remdesivir, an adenosine analog that incorporates into RNA and causes premature termination, is a promising antiviral agent, with preliminary reports suggesting clinical benefit in adult patients. It is available through compassionate use, as well as emergency authorization use, for neonates. Another promising option is convalescent plasma obtained from patients who have recovered from COVID-19 infection with detectable antibodies. It is available as part of clinical trials in many centers. At the beginning of the outbreak, hydroxychloroquine was used as a first-line agent, however, there is insufficient data to suggest any benefit. In addition, COVID-19 pneumonia is associated with a severe inflammatory response, including high pro-inflammatory cytokines such as interleukin-6 (IL-6). An IL-6 monoclonal antibody, tocilizumab, is being used as part of clinical trials with benefit.

## Conclusions

Based on the limited evidence available at this time, the probable mode of transmission of neonatal COVID-19 appears to be horizontal while vertical transmission is a possibility in early-onset infections. Fever is one of the main presenting symptoms of neonatal COVID-19 and its testing should be considered in the evaluation of a febrile infant presenting to the emergency department. Infected infants can shed the virus through the respiratory tract as well as in stool. Thereby, caregivers should take caution with good hand hygiene, particularly when changing diapers. Thus, prompt recognition of illness in this population is essential to limit further transmission in the community. Finally, it is important to understand that these conclusions are based on limited data and our understanding will continue to evolve.
